# The Development and Validation of a Short Form of the Forbearance Scale

**DOI:** 10.3389/fpsyg.2021.686097

**Published:** 2021-07-15

**Authors:** Man Yee Ho, Siya Liang

**Affiliations:** Department of Social and Behavioural Sciences, City University of Hong Kong, Kowloon, Hong Kong

**Keywords:** forbearance, scale development, factor analysis, scale validation, assessment

## Abstract

The Forbearance Scale (FS) is a 16-item self-report measure of forbearance. In this study, we examined the psychometric properties of the FS subscale and composite scores and developed a 9-item short form of the measure (FS-SF 9). A sample of 1,137 participants was drawn from community, NGO, and college settings. The sample was split into a derivation sample (*n* = 567) and a validation sample (*n* = 570). Exploratory factor analyses of the derivation sample data were used to select short-form items. Using the validation sample, confirmatory factor analyses were used to assess fit for proposed item-to-factor assignments. The results of the confirmatory factor analyses supported that the FS-SF 9 had a theoretically congruent factor structure and that all the subscale and composite scores displayed high internal consistency. Correlations with scores from established measures of a lack of forgiveness and emotion regulation also supported the validity of the FS-SF 9. Our data suggest that the FS-SF 9 subscales and composite score retained the psychometric strengths of their longer FS counterparts. Overall, the short form of the FS provides a brief assessment of the construct measured by the full form. Theoretical and practical applications are discussed.

## Introduction

Conflicts are an inevitable aspect of all relationships. If conflicts are not handled properly, negative responses to them can take an emotional toll on mental health (Seidi and Jaff, 2019). A lack of forgiveness is considered a negative response to conflicts in close relationships. Research has shown that forgiveness is linked to positive mental health outcomes. For example, Chung (2016) found that a lack of forgiveness was related to depressive symptoms. Toussaint et al. (2016) indicated that forgiveness was associated with a decrease in stress, which in turn was related to a decrease in psychiatric symptoms.

As the world becomes more globally connected, nations are becoming increasingly multicultural. Multicultural personality traits appear to be important predictors of intercultural competence and how successful an individual will be in adapting to multicultural situations. According to Ho (2020), forbearance is a closely related concept to forgiveness that seems to be more applicable and culturally relevant in Asian societies. Forbearance is conceptualized as a coping strategy that facilitates the maintenance of interpersonal harmony (Wei et al., 2012). Unlike forgiveness, the construct of forbearance has been largely overlooked, and well-validated measures of forbearance are scant in the literature.

The construct of forbearance is of considerable interest to personality assessment, as it is essential to positive relationships, particularly in relation to maintaining social harmony and repairing personal relationships. Forbearance can also be viewed as an attempt to ameliorate a situation to make it more tolerable for others, and it is considered a coping strategy for settling interpersonal disputes (Wei et al., 2012).

In collectivistic cultures, forbearance is a highly valued virtue representing a high level of benevolence and kindness. In Chinese philosophy—Confucianism—forbearance is considered a virtue in which individuals embrace the qualities of kindness and tolerance while considering others' perspectives (Han and Zhang, 2018). People from collectivistic cultures promote and exercise forbearance more often in the face of provocation as a means of maintaining positive social relationships (Yeh et al., 2006). Additionally, forbearance is a significant predictor of well-being, including happiness, family harmony, and teamwork in Hong Kong (Ho, 2020). Therefore, the assessment of forbearance becomes important, and it deserves further scientific investigation.

Existing forbearance measures usually assess a unidimensional construct of forbearance. For example, the Forbearance subscale of the Collectivistic Coping Styles Measure (CCSM; Moore and Constantine, 2005) assesses the tendency to minimize or conceal problems or concerns so as not to trouble or burden others, but it does not capture the complexity and multidimensional nature of the construct.

The original 16-item Forbearance Scale (FS) developed by Ho (2021)^1^ is a self-report instrument that measures trait levels of forbearance. It was designed to assess the cognitive, emotional, and behavioral components of the construct. A recent study has developed and validated a four-factor model of the FS in the context of Hong Kong (Ho, 2021)^1^. This FS has four subscales (of four items each): (1) overlook others' misdeeds, (2) tolerance and acceptance, (3) emotional calmness, and (4) self-restraint. Scores from the four subscales are also designed to be combined into a composite score, which indicates an overall level of forbearance.

Previous literature suggest that the concept of forbearance implies the meaning of overlooking others' misdeeds and minor imperfections (Foxe et al., 2015; Pianalto, 2016). Forbearance is also defined as tolerance in the face of provocation (American Heritage Dictionary, 2011). A forbearing person often responds in a calm and sensible way at a time when they would have the right to be very upset or angry (Cobuild Advanced English Dictionary, 2016). Furthermore, based on Fortner's (2014) definition, forbearance entails self-restraint, self-control, and self-discipline. Regarding the specific dimensions in this study, *overlooking others*' *misdeeds* refers to not paying too much attention to the perceived faults of others. *Tolerance and acceptance* denote allowing and supporting others' practices, opinions, and beliefs, especially if one does not necessarily agree with them. *Emotional Calmness* refers to the moderation of one's emotions and the ability to remain calm under interpersonal stress. *Self-Restraint* reflects one's capacity to self-regulate and control one's behavior.

To date, the psychometric properties of FS scores have been examined in two college student samples in Hong Kong (Ho, 2020). Exploratory and confirmatory factor analyses found that the FS has a theoretically congruent factor structure consisting of four first-order factors. All the subscale and composite scores had high internal reliability and correlations with other construct validity measures (Ho, 2020).

Although the previously mentioned results are promising for the usefulness of the FS and the length of the FS is not excessive, some researchers and practitioners may want to have a shorter measure to use in a study that incorporates multiple psychological instruments. Our goal was to develop an FS short form with the same structure as the full form that also showed strong agreement with the full form in terms of scores. We believe the development of a short form of the FS would increase the utility of the instrument as a personality assessment tool while making practical implementation less burdensome and more time effective. Furthermore, the development of a short form of the FS has substantial research implications; in particularly, this brief FS provides additional evidence for the multidimensional nature of forbearance. The shortened version of the FS is more culturally appropriate in collectivistic cultures, which emphasize group cohesion and social harmony. In addition, the three-factor model of the short form of the FS also advances our conceptual understanding of forbearance.

In this study, exploratory and confirmatory factor analyses were used to develop a short form with a structure similar to the original FS. Moreover, a validation sample confirmed the goodness of fit of the short version. Furthermore, to examine the content and construct validities of the short form, item-scale correlations, criterion-related indicators, and interrelationships with measures of emotion regulation and a lack of forgiveness were evaluated.

## Method

### Participants

This study used a pooled sample of participants enrolled in three extant studies. This includes a college student sample from a study of personality and character, a sample of dating or married adults from a study of personality and romantic relationships, and a sample of parents from a study of personality and parent-child relationships.

#### College Student Sample

Participants were 184 college students (39.7% male; 60.3% female) from a local university in Hong Kong. All the students were between 18 and 24 years old (*M* = 19.40, *SD* = 1.53). The majority of participants (73.9%) in this sample reported not being affiliated with a religion, 20.7% were Christian, and 5% each were Catholic and Buddhist. Participants were given course credit for participating in this research.

#### Dating or Married Adult Sample

This sample consisted of 817 dating or married adults (34.9% male; 65.1% female) who had been involved in a heterosexual romantic relationship for at least 6 months (*M* = 79.49, *SD* = 95.36). Participants were recruited from NGOs, religious communities, social media, and advertising on mass public transport. Roughly one-third of the participants in this sample (34.1%) identified themselves as married, and 65.9% identified as single. The majority of participants (74.3%) held a bachelor's degree or above, 16.8% held a diploma or associate degree, and 8.9% had a secondary school diploma or below. More than half of the participants (69.4%) reported not being affiliated with a religion, 21.9% were Christian, 5% were Catholic, 2.9% were Buddhist, and the rest (0.8%) did not report a religion. The participants each received a HK$100 (~US$12.80) supermarket coupon as compensation for their time.

#### Parent Sample

In total, 136 parents of children with special education needs (SEN; 7.4% male and 92.6% female) were recruited from a local NGO. The parents' ages ranged from 27 to 69 years (*M* = 41.24, *SD* = 6.91). The majority of participants (96.3%) in this sample were parents; however, 3.6% of them were grandparents. More than half of the participants (64%) had a secondary school diploma or below, 16.9% had a diploma or associate degree, and 19.1% had a bachelor's degree or above. The majority (58.6%) reported not being affiliated with a religion, 30.1% were Christian, 6.8% were Buddhist, 2.3% were Catholic, 1.5% were Taoist, and the remaining 0.7% did not report their religion. The participants each received a HK$50 (~US$6.40) supermarket coupon as compensation for their participation.

#### Derivation and Validation Samples

All the participants were pooled into a single dataset (*N* = 1,137). The SPSS (Version 27) program was used to randomly split each sample in the dataset to create a composite derivation sample (*n* = 567; 16.2% from the student sample, 71.8% from the dating or married adult sample, and 12% from the parents of children with SEN) and a composite validation sample (*n* = 570; 16.1% from the student sample, 71.9% from the dating or married adult sample, and 11.9% from the parent sample). The two samples did not differ significantly in terms of gender (χ^2^_(1)_ = 1.78, *p* = 0.184), age (*t*_(1135)_ = 0.18, *p* = 0.855), education (χ^2^_(2)_ = 2.59, *p* = 0.274), or religion (χ^2^_(4)_ = 12.19, *p* = 0.702). In addition, no significant difference was found in the FS-16 scores between the derivation sample and validation sample (*ps* > 0.07).

### Measures

#### Forbearance Scale

The FS (FS; Ho, 2021)^1^) consists of 16 items that measure a person's level of patient self-control in an interpersonal context. The FS, as mentioned above, is divided into four subscales: overlooking others' misdeeds, tolerance and acceptance, emotional calmness, and self-restraint. Respondents were asked to rate items on a 6-point Likert scale ranging from 1 (*strongly disagree*) to 6 (*strongly agree*). All scores were summed to obtain a total score, with higher scores indicating a higher level of forbearance. The FS has demonstrated very good internal consistency (α = 0.84), good construct validity, and was moderately correlated with forgiveness in Hong Kong samples (Ho, 2020). The total FS score yielded relatively good internal consistency (α = 0.80 in a derivation sample) in the current study.

#### Emotion Regulation Questionnaire

The Emotion Regulation Questionnaire (ERQ; Gross and John, 2003) is a 10-item self-report measure of two emotion regulation strategies: (1) Cognitive Reappraisal and (2) Emotional Suppression. Respondents rate items on a 7-point Likert scale that ranges from 1 (*strongly disagree)* to 7 (*strongly agree*). In the current study, ERQ demonstrated acceptable internal consistency, α = 0.61 (Cognitive Reappraisal) and α = 0.70 (Emotional Suppression).

#### Transgression-Related Interpersonal Motivations Inventory Within a Chinese Context

The Transgression-Related Interpersonal Motivations Inventory within a Chinese Context (C-TRIM; Wong et al., 2014) is a 12-item scale that represents the opposite of forgiveness (i.e., avoidance and revenge). Respondents were asked to respond to items using a 5-point Likert scale ranging from 1 (*strongly disagree*) to 5 (*strongly agree*). The total score is obtained by summing across all the items. Higher scores indicate less forgiveness. In the current study, the internal consistency of the C-TRIM was α = 0.92.

### Procedure

#### Data Collection

The data collection methods varied by sample. Participants in the college student sample learned of the study through a university-wide broadcast of email messages, posters, and advertisements on campus, and then completed an online survey. Participants in the dating or married adult sample learned of the study through NGOs, religious communities, social media, and advertising on mass public transport and were given access to the online survey. Participants in the parent sample learned of the study through a local NGO and completed a paper-and-pencil self-administered questionnaire. The procedures, protocol, and informed consent in the study were approved by the institutional ethics review committee of the City University of Hong Kong (Reference No.: 11610817). Informed consent was obtained from all participants prior to their participation in the study.

#### Short Form Item Selection

We developed the short form of the Forbearance Scale (FS-SF) with a mixed method approach that involved exploratory factor analysis (EFA), classical test theory, and expert input. Using the derivation sample, a maximum likelihood (ML) extraction with direct oblimin rotation (Schmitt et al., 2018) was performed. In seeking a simple structure within the pattern matrix, our goal was to reduce the number of candidate items in the final short form based on three criteria: first, all item loadings are interpretable (>0.40); second, each factor must have a minimum of three items; and third, there is an absence of problematic cross-loadings (>0.20). This is consistent with the guidance from the literature (Raubenheimer, 2004; Tabachnick and Fidell, 2007).

A series of EFAs were conducted to further explore the factor structure of the FS. To identify core items of the scale, we first entered all 16 items and then removed them one at a time. Items with interpretable loadings and acceptable cross-loadings in the pattern matrix were retained to achieve the best-fitting model. To examine whether additional items would generate interpretable loadings, we readded previously removed items one by one. If the additional item yielded a larger loading on a factor compared to the previously retained items, then the newly added item was also retained; otherwise, it was not. This process was repeated until all the items were given multiple chances to enter the models.

To assess the internal consistency of the short form of the FS, both reliability and correlation analyses were performed. We adopted a classical test theory-based reliability matrix (Ponterotto and Ruckdeschel, 2007) in the reliability testing. If the Cronbach's alpha scores of the scale and subscales were lower than 0.65, items were reexamined, and alternative items that contributed substantially to increasing the Cronbach's alpha scores were reentered into the model. This was done until the Cronbach's alpha scores of the resulting scale and subscales were all above 0.65. In the correlation analysis, any factor with a non-significant correlation with other factors in the instrument was removed, and items within this factor that had weak or no correlations with items from other factors and the total scores were also removed (this was seen as an indication that a subscale was “too distinct” to be grouped with the others as part of the overall measure of forbearance). Nine items were identified in the optimal factor structure for the shortened version of the FS; the EFA was repeated on the remaining items.

#### Final Subscale Analyses

To reduce the subscale length while retaining adequate psychometric properties through replicable CFA procedures and maintaining a minimum Cronbach's alpha value of 0.70, the final item selection was based on high factor loadings and lower levels of negative skew (<|1.50|).

A parallel analysis (PA; Zwick and Velicer, 1986) using O'Connor's (2000) SPSS syntax was conducted to determine the factor structure of the scale. We used permutations of the derivation sample to generate 1,000 different samples for this analysis. Actual eigenvalues were compared with the 99th percentile estimates from the permutation samples.

Using the validation sample, we then subjected the short-form FS to the CFA procedures. Given the theoretical and practical importance of modeling a total score and subscales scores in the structure of the short form of the FS, correlated three-factor, higher order, and bifactor models were examined with the validation sample. CFA models were generated using ML estimation (Li, 2016). Model goodness of fit was evaluated via the root mean square error of approximation (RMSEA) and three incremental fit indexes: the comparative fit index (CFI), the Tucker-Lewis index (TLI), and the standardized root mean square residual (SRMR). We adopted a cluster of goodness-of-fit criteria commonly used in psychological research (Wang and Wang, 2012). Specifically, CFI and TLI values >0.90 indicated an adequate fit, as did RMSEA and SRMR values <0.08 (Wang and Wang, 2012; Schmitt et al., 2018).

#### Validity

Content validity was examined by calculating the item-scale correlation coefficients. Construct validity was reflected in the model fit statistics after conducting CFA, along with the composite validity (CR) and average variance extracted (AVE), as recommended by Hair et al. (2010), a CR valued above 0.60 and AVE valued above 0.50 demonstrated convergent validity (Fornell and Larcker, 1981), and the square root of the AVE being higher than any of the correlations involving the designated factor within the structure indicated good divergent validity. External construct validity was explored using the dating or married adult sample. Pearson correlations among the ERQ, C-TRIM, and FS-SF and stepwise hierarchical regressions were performed because forbearance is closely linked to emotional expression, negative vengefulness, and avoidant motives (Wong et al., 2014; Lin, 2016).

## Results

Mplus 8.4 was used to perform the EFAs and CFAs, and SPSS 27 was used for all the other analyses. All the FS items were reasonably normally distributed (maximum skewness = 0.47 and maximum kurtosis = 0.76).

### Exploratory Factor Analyses: Derivation Sample

The results of the EFA on the original 16 items are shown in Table 1, Item 3 with uninterpretable factor loading (<0.40) and Item 2 with problematic cross-factor loading (>0.20) were first eliminated. Items that yielded the smallest loading on a factor were removed one at a time to balance the length of each subscale. After a series of EFAs, a four-factor model (12 items with three items per factor) was first identified. The four-factor structure accounted for 69.92% of the variance, and the resultant eigenvalues were as follows: overlook others' misdeeds, 4.02 (33.51%); self-restraint, 1.77 (14.73%); emotional calmness, 1.37 (11.43%); and tolerance and acceptance, 1.23 (10.26%). The item loadings in the pattern matrix ranged from 0.72 to 0.90 for overlook others' misdeeds, 0.53–0.71 for self-restraint, 0.61–0.98 for Emotional Calmness, and 0.64–0.68 for Tolerance and Acceptance. The communalities (after extraction) ranged from 0.55 (Item 9) to 0.85 (Item 14). Although Items 7, 9, 10, 13, and 14 had small cross-loadings, these were not problematic (<0.20 and not within 0.20 of their primary loadings).

**Table 1:** Results of exploratory factor analysis on the original Forbearance Scale.

**Item**	**Factor loadings**			
	**EC**	**OOM**	**TA**	**SR**
1. I can stay calm when others misunderstand me.	**0.516**	**0.117**	0.081	0.035
4. I feel at peace even if others are being unreasonable.	**0.673**	0.063	0.010	0.052
13. I remain calm when others are rude to me.	**0.819**	−0.028	0.062	−0.020
14. I stay unmoved when others insult me.	**0.940**	−0.012	−0.050	−0.011
2. I don't care much about others' faults.	**0.278**	**0.464**	−0.013	−0.035
6. It's easy for me to forget others' faults.	0.009	**0.708**	0.005	−0.029
8. I don't take others' faults to heart.	0.001	**0.885**	0.007	0.022
12. I overlook others' mistakes.	0.016	**0.785**	0.054	0.004
5. I listen to others' opinions when I disagree with them.	0.004	0.006	**0.707**	0.014
7. I accept other's opinions even if I disagree with them.	−0.014	**0.170**	**0.646**	0.023
11. I try to tolerate others' opinions even if I disagree with them.	0.061	−0.055	**0.652**	−0.027
16. I am inclusive to beliefs I don't agree with.	0.016	**0.106**	**0.615**	−0.018
3.^*^I defend myself immediately if others put the blame on me.	0.019	0.034	−0.105	**0.358**
9.^*^I retaliate when others ridicule me.	0.013	0.070	**−0.140**	**0.615**
10.^*^I directly express my negative emotions when my opinions aren't accepted.	−0.094	−0.034	0.062	**0.580**
15.^*^I lose control of my emotions when provoked by others.	0.034	−0.079	0.031	**0.648**

*Loadings that are significant at 5% level were shown in bold. EC, Emotional Calmness; OOM, Overlook Others' Misdeeds; TA, Tolerance and Acceptance; SR, Self-Restraint*.

* *Item was reverse coded*.

### Agreement With Full-Form Scores, Subscale Intercorrelations, and Internal Consistency

Table 2 presents the bivariate correlation matrix inclusive of the original FS-16 and the short form FS-SF 12. Each subscale correlated highly and significantly with its corresponding long subscale, ranging from 0.94 (Self-Restraint) to 0.97 (Emotional Calmness). From these data, it was established that the new short form was performing like the original-length version.

**Table 2:** Correlation matrix of the FS-16 and FS-SF 12 subscales.

	**FS-16**			**FS-SF12**			
	**OOM**	**TA**	**SR**	**EC**	**OOM**	**TA**	**SR**
FS-16							
EC	0.53^**^	0.39^**^	0.008	0.97^**^	0.45^**^	0.38^**^	0.006
OOM		0.45^**^	−0.06	0.50^**^	0.97^**^	0.43^**^	−0.06
TA			−0.06	0.36^**^	0.44^**^	0.97^**^	−0.06
SR				0.008	−0.07	−0.07	0.94^**^
FS-SF 12							
EC					0.44^**^	0.36^**^	0.003
OOM						0.42^**^	−0.05
TA							−0.05

*FS-16, Forbearance Scale (16-item); FS-SF 12, Forbearance Scale-Short Form (12-item); EC, Emotional Calmness; OOM, Overlook Others' Misdeeds; TA, Tolerance and Acceptance; SR, Self-Restraint*.

** *Correlation is significant at the 0.01 level*.

The low to moderate correlations among the subscales of the FS-SF supported the relative independence of the three factors (i.e., emotional calmness, overlooking others' misdeeds, and tolerance and acceptance). However, no significant correlation was found between Self-Restraint and the other factors (*rs* < |0.05|), which did not support the construct validity of the four-factor model. The items from Self-Restraint were also weakly correlated with items from other factors (*rs* < 0.11) and with the total score (*rs* < 0.29, see Table 3). The results of the EFA and the correlation analysis suggest that the self-restraint subscale was different from the subscales for overlooking others' misdeeds, tolerance and acceptance, and emotional calmness.

**Table 3:** Correlation matrix of SR subscale and EC, OOM, TA subscales and total score of FS-12.

**Item no**.		**EC**			**OOM**			**TA**			**Total score**
		**4**	**13**	**14**	**6**	**8**	**12**	**5**	**7**	**11**	
SR	9	0.04	−0.03	0.03	−0.03	0.01	−0.01	−0.08	−0.04	−0.11^*^	0.27^**^
	10	−0.04	−0.03	−0.07	−0.05	−0.05	−0.04	0.01	0.01	−0.06	0.26^**^
	15	0.07	0.04	0.02	−0.05	−0.04	−0.04	−0.00	−0.03	0.02	0.29^**^
	Subscale score		0.00			−0.05			−0.05		0.30^**^

*SR, Self-Restraint; EC, Emotional Calmness; OOM, Overlook Others' Misdeeds; TA, Tolerance and Acceptance; FS-12, 12-item Forbearance Scale.*.

* *p < 0.05*;

** *p < 0.01*.

In our study, the self-restraint subscale seemed to be different from the overlooking others' misdeeds, tolerance and acceptance, and emotional calmness subscales in how it measured forbearance. Upon reflection, it seemed that the self-restraint subscale measured forbearance from a behavioral intention perspective, in which a person was expected to act less aggressively when he or she experienced negative feelings, while the other three subscales measured forbearance from a cognitive and emotional perspective, in which an individual was supposed to be more condoning, more tolerant, and emotionally calm when being provoked. The findings of the current study could mean that self-restraint is weakly associated with the cognitive and emotional aspects of forbearance.

In understanding the concept of forbearance, the inclusion of a self-restraint factor in the construct of forbearance is still debatable. Consistent with Lin's (2014) conceptualization, forbearance can be defined as an emotional expression. Moreover, a synonym for forbearance is tolerant perseverance (Neenan and Dryden, 2020). The cognitive and emotional components of forbearance seem to be more prominent for understanding the construct of forbearance. In the current study, a three-factor model fit the data better than the four-factor model. Thus, the self-restraint subscale (items 9, 10, and 15) was eliminated from the short form in this study.

Table 4 summarizes the scale descriptors and alpha coefficients for the original FS-16 and the FS–SF 9. We used the 9-item version of the FS-SF; all its subscales (overlook others' misdeeds, emotional calmness, and tolerance and acceptance) were within acceptable ranges and were moderately correlated with one another. Agreement between each of the three subscales and the total score was strong. Cronbach's alpha values ranged from 0.73 to 0.85. All the alpha coefficients were above 0.70, indicating good internal consistency for the FS-SF 9.

**Table 4:** Descriptive comparisons of FS-16 and FS-SF 9.

	***M***	***SD***	**Skewness**	**α**	**^a^ interpretation^**a**^**
FS-16					
EC	3.53	0.94	0.11	0.85	Good
OOM	3.93	0.92	−0.09	0.84	Good
TA	4.39	0.66	−0.30	0.78	Moderate
SR	3.38	0.81	−0.17	0.63	Fair
Total score	3.81	0.54	−0.08	0.80	Good
FS-SF 9					
EC	3.54	0.98	0.10	0.85	Good
OOM	4.00	0.98	−0.17	0.84	Good
TA	4.39	0.69	−0.27	0.73	Moderate
Total score	3.98	0.69	0.17	0.84	Good

*FS, Forbearance Scale; FS-SF 9, Forbearance Scale-Short Form (9-item); EC, Emotional Calmness; OOM, Overlook Others' Misdeeds; TA, Tolerance and Acceptance; SR, Self-Restraint*.

a *Qualitative interpretations of coefficient alpha sufficiency are based on the classical test theory-anchored “Reliability Matrix for Estimating Adequacy of Internal Consistency Coeffificients” where the range is Unsatisfactory, Fair, Moderate, Good, and Excellent (Ponterotto and Ruckdeschel, 2007, Table 3, p. 1003)*.

### Parallel Analysis

Nine items were eventually identified for the short form of the FS. The parallel analysis suggested three factors with an eigenvalue of 1.23 necessary to retain a factor; thus, all three factors were retained. We repeated the EFA while specifying a 9-item, three-factor extraction. The communalities and factor loadings from the pattern matrix are shown in Table 5. The three-factor model accounted for 66.38% of the variance, and the resultant eigenvalues were as follows: overlook others' misdeeds, 4.02 (40.15%); emotional calmness, 1.39 (13.89%); and tolerance and acceptance, 1.24 (12.35%). Item loadings in the pattern matrix ranged from 0.73 to 0.90 for overlooking others' misdeeds, 0.63–1.00 for emotional calmness, and 0.64–0.67 for tolerance and acceptance. The communality estimates (after extraction) ranged from 0.66 (Item 5) to 0.85 (Item 14). Although Items 7 and 14 had small cross-loadings, these were not problematic (<0.20 and not within 0.20 of their primary loadings).

**Table 5:** Exploratory factor analysis item loadings from pattern matrix, mean, standard deviation, skewness, and communality estimates for three-factor FS-SF 9.

**Item**	**Factor**			***M***	***SD***	**Skew**	**Comm**
	**EC**	**OOM**	**TA**				
4. I feel at peace even if others are being unreasonable.	**0.63**	0.07	0.02	3.45	1.16	0.18	0.67
13. I remain calm when others are rude to me.	**0.81**	0.009	0.02	3.73	1.05	−0.12	0.79
14. I stay unmoved when others insult me.	**1.00**	−0.005	**−0.12**	3.44	1.16	−0.03	0.85
6. It's easy for me to forget others' faults.	0.002	**0.73**	−0.01	4.01	1.25	−0.35	0.73
8. I don't take others' faults to heart.	−0.007	**0.90**	−0.008	4.00	1.05	−0.22	0.81
12. I overlook others' mistakes.	0.02	**0.78**	0.04	3.98	1.06	−0.21	0.76
5. I listen to others' opinions when I disagree with them.	0.02	0.03	**0.67**	4.45	0.87	−0.47	0.66
7. I accept other's opinions even if I disagree with them.	−0.07	**0.18**	**0.65**	4.40	0.83	−0.35	0.66
11. I try to tolerate others' opinions even if I disagree with them.	0.01	−0.04	**0.64**	4.28	0.84	−0.31	0.66

*Loadings that are significant at 5% level are shown in bold. FS-SF 9, short-form of Forbearance Scale (9-item); EC, Emotional Calmness; OOM, Overlook Others' Misdeeds; TA, Tolerance and Acceptance; Comm, communality estimate*.

### Confirmatory Factor Analyses: Validation Sample

First, a correlated three-factor model was tested. We assigned Items 6, 8, and 12 to Factor 1 (overlook others' misdeeds) with their respective loadings of 0.64, 0.80, and 0.76. Items 4, 13, and 14 were assigned to Factor 2 (emotional calmness) with their respective loadings of 65, 0.86, and 0.93. Items 5, 7, and 11 were assigned to Factor 3 (tolerance and acceptance) with their respective loadings of 0.72, 0.69, and 0.64. The overall model was significant, *X*^2^(24) = 82.42, *p* < 0.001, CFI = 0.97, TLI = 0.96, SRMR = 0.042, RMSEA = 0.065, 90% CI = [0.050, 0.081], demonstrating that the short form of the FS could be used as a multidimensional instrument.

Second, a hierarchical model was examined and yielded a good fit, *X*^2^(24) = 82.42, *p* < 0.001, CFI = 0.97, TLI = 0.96, SRMR = 0.043, RMSEA = 0.065, 90% CI = [0.050, 0.081], with emotional calmness loading 0.60, Tolerance and Acceptance loading 0.80, overlooking others' misdeeds loading 0.69 on the second-order factor, Forbearance, indicating that the sum score of the three factors was reliable.

Third, the bifactor model was further examined. Model fit statistics also suggested good fit, *X*^2^(18) = 34.60, *p* < 0.05, CFI = 0.99, TLI = 0.97, SRMR = 0.027, RMSEA = 0.040, 90% CI = [0.019, 0.060], implying that the short form of the FS maintained a unidimensional structure.

### Validity

#### Content Validity

Pearson correlation coefficients were calculated between each of the final items of the FS-SF 9 and its corresponding subscale. The scale demonstrated good item-scale correlations (*rs* > 0.46), and the ranges of coefficients were as follows: emotional calmness ranged from 0.53 to.81, overlooking others' misdeeds ranged from 0.47 to 0.83, and tolerance and acceptance ranged from 0.47 to 0.84.

#### Construct Validity

The CFA previously reported supported the construct validity of the FS-SF 9. The pattern of inter-scale correlations and CR and AVE of the latent variable reported in Table 6 provided additional supports for the FS-SF 9's construct validity. Though the AVE of Tolerance and Acceptance was 0.43, the CR of this latent variable was 0.69, which was still deemed as adequate for the convergent validity of the construct by Fornell and Larcker (1981).

**Table 6:** FS-SF 9 intercorrelation matrix, CR, AVE, square root of AVE of latent variable, and correlations between FS-SF 9 and emotional regulation, unforgiveness.

				**ERQ**		
**FS-SF 9**	**EC**	**OOM**	**TA**	**Cognitive reappraisal**	**Expressive suppression**	**C-TRIM**
Total score	0.80^**^	0.78^**^	0.75^**^	0.25^**^	0.19^**^	−0.14^**^
EC	/	/	/	0.16^**^	0.23^**^	−0.04
OOM	0.35^**^	/	/	0.18^**^	0.10^**^	−0.15^**^
TA	0.43^**^	0.43^**^	/	0.27^**^	0.11^**^	−0.16^**^
CR	0.86	0.85	0.69			
AVE	0.68	0.65	0.43			
Square root of AVE	0.82	0.81	0.66			

*FS-SF 9, Forbearance Scale-Short Form (9-item); ERQ, Emotion Regulation Questionnaire; EC, Emotional Calmness; OOM, Overlook Others' Misdeeds; TA, Tolerance and Acceptance; C-TRIM, Transgression-Related Interpersonal Motivations Scale within a Chinese context; CR, Composite Reliability; AVE, Average Variance Extracted*.

** *Correlation is significant at the 0.01 level*.

#### External Construct Validity

To explore construct validity with external indicators, we used the dating and married adult sample and calculated scores for the 9-item version of the forbearance scale and each subscale. The zero-order correlations are reported in Table 6. The results showed that all the subscales were positively correlated with cognitive reappraisal and expressive suppression, while revealing a significant negative correlation between the overlooking others' misdeeds subscale and the C-TRIM (a measure of less forgiveness), as well as between the tolerance and acceptance subscale and the C-TRIM. However, the correlation between the emotional calmness subscale and the C-TRIM was not statistically significant. The results from the hierarchical regressions predicting emotion regulation and a lack of forgiveness can be found in Table 7.

**Table 7:** Hierarchical regressions predicting emotion regulation and unforgiveness.

**Dependent**	**Predictor**	**β**	***t***	***R***	***R^2^***	***F***	***df***
CR	TA	0.27	7.91^**^				
			Final model	0.27	0.07	62.59	815
ES	EC	0.23	6.83^**^				
			Final model	0.23	0.05	46.70	815
C-TRIM	OOM	−0.10	−2.47^*^				
	TA	−0.12	−3.10^**^				
			Final model	0.18	0.03	14.19	814

*CR, Cognitive Reappraisal; ES, Emotional Suppression; C-TRIM, Transgression-Related Interpersonal Motivations Scale within a Chinese context; EC, Emotional Calmness; OOM, Overlook Others' Misdeeds; TA, Tolerance and Acceptance.*

* *Correlation is significant at the 0.05 level*.

** *Correlation is significant at the 0.01 level*.

## Discussion

The aim of this study was to generate a shorter measure of forbearance. With that goal in mind, we developed a 9-item short form of the FS and examined the psychometric properties of the measure with both the derivation and validation samples. Our findings indicate that the FS-SF 9 was rigorous in terms of its internal structure and suitability for measuring forbearance. The results of both the EFA and CFA suggest that the short form of the FS was a valid and reliable measure. The score agreement was adequate to strong at all levels (the overall FS-SF 9 score and the scores for the overlooking others' misdeeds, tolerance and acceptance, and emotional calmness subscales). All the short-form subscales were internally consistent and unidimensional. The results of the CFA suggested an excellent fit of the measurement model, and all the FS-SF 9 subscales showed good reliability.

The investigation of the structure of the FS-SF 9 with CFA demonstrated that the scale could be used as both a unidimensional and multidimensional instrument. The scores of the subscales and the whole scale are reliable and can be used as indicators of forbearance.

The construct validity findings provide initial support for the FS-SF 9 as a measure of forbearance. Content validity implied consistency among the items within each factor. The exploration of the convergent and divergent validity of the FS-SF 9 yielded additional support for the maintenance of the current structure. Additionally, the overall FS-SF 9 was positively associated with emotion regulation and negatively associated with a lack of forgiveness. Although there was some variation in magnitudes, the subscales of the FS-SF 9 were also correlated with the convergent and discriminate measures in similar directions.

The CFA item elimination process resulted in excluding seven of 16 items. The items that were selected in this study were interpretable and coincided with the established structure of the original instrument. Only one item (the one with the lowest loading) was excluded in each subscale. The three subscales seemed to be reasonably robust, with reliable factor loadings and few secondary loadings while maintaining high correlations (*rs* > 0.95) with the original subscales. This was especially the case with the overlooking others' misdeeds subscale, in which all loadings were above 0.70 with no secondary loadings.

Although it is not a primary focus of this article, the results of the regression analyses suggest that some subscales might be more uniquely predictive of specific indicators than others. For example, the tolerance and acceptance subscale was positively linked to cognitive reappraisal. The emotional calmness subscale was positively correlated with emotional suppression. The tolerance and acceptance subscale and the overlooking others' misdeeds subscale were both predictive of a lack of forgiveness. The overall FS-SF 9 is likely to be most useful in personality assessment, as the present findings suggest that some subscales might show more specific associations with different aspects of emotion regulation and a lack of forgiveness.

In the shortened form, the self-restraint subscale was removed due to differences found between it and the other three subscales. This could be because it is a distinct dimension from the other three that may more closely reflect the cognitive and emotional dimension of forbearance. Unlike in the work of Ho (2020), in the current study, the concept of forbearance was mainly derived from cognitive and emotional perspectives embedded in broader cultural contexts. In a collectivistic culture, people tend not to express (and correspondingly, have the need to suppress) negative emotions and thoughts (e.g., anger or revenge motives) in response to transgressions or hurt for the sake of social harmony (Matsumoto et al., 2008; Wei et al., 2012). Although forbearing persons may experience a transformation of cognition (e.g., overlook others' misdeeds) and emotions (e.g., emotional calmness), they may not necessarily have negative emotions or thoughts that require the exercise of control (e.g., self-restraint) in the face of interpersonal conflicts. However, additional research in examining these distinctions need to be performed in the future.

It is of interest if a dimension of behavioral intention is different from dimensions of cognition and emotion in the understanding of forbearance. In alignment with previous research, the findings of this study add support to the notion that the cognitive and emotional components of forbearance are crucial and universal across cultures (Lin, 2014; Kobylińska and Kusev, 2019). Based on the psychometric properties of the FS-SF 9, instead of the original four-factor structure, a three-factor model was identified in this study. Moreover, in consideration of its adaptability of the forbearance measure in wider cultural contexts, it is recommended that items from the self-restraint subscale be removed form short forms of the FS. Again, additional research is needed to substantiate our reasoning behind this and how well it generalizes in similar contexts.

Even though the short form achieved many of our aims, there are several limitations to our investigation. First, the findings of this study may not generalize to other cultures that differ in substantial ways. Hong Kong's culture is a mixture of foreign cultural influences and traditional Chinese values (Hinsbergh, 2020); using other diverse cultural groups in research should advance and deepen our understanding of forbearance. Future research might seek to discover the extent of cultural variation in forbearance (vs. its more universal aspects) by utilizing various samples. Additionally, regarding the analytical approach, the evaluation of the subscales was mainly based on their factor structure. This approach might not be optimal for assessing the quality of the FS-SF 9 subscales. Further investigation might explore alternative approaches, such as item-response theory, for generating different item sets for the short-form subscales. Furthermore, social desirability response bias is another limitation that suggests a need for a cautious interpretation of the results. The results presented in this study were limited to cross-sectional correlations among self-report measures. Future studies would likely benefit from including the ratings of an individual by others and physiological and behavioral measures.

In conclusion, this study makes a useful contribution to the field of psychology in the area of forbearance, as it represents the first attempt to develop and validate a shorter form of the FS. Theory and research on multicultural personality traits have been identified as a priority across a number of disciplines (Ponterotto, 2010). As multicultural personality traits are expected to be adaptive to individuals living in culturally heterogeneous societies, measurements aimed at assessing such traits could prove to be salient to researchers and practitioners. It is hoped that the development and validation of the FS-SF 9 will ease and accelerate cross-cultural research in this important area of study.

## Data Availability Statement

The raw data supporting the conclusions of this article will be made available by the authors, without undue reservation.

## Ethics Statement

The studies involving human participants were reviewed and approved by Human Subjects Ethics Sub-Committee at City University of Hong Kong. The patients/participants provided their written informed consent to participate in this study.

## Author Contributions

MH and SL have directly participated in the planning, execution, and analysis of this study. Both authors have read and approved the final version submitted.

## Conflict of Interest

The authors declare that the research was conducted in the absence of any commercial or financial relationships that could be construed as a potential conflict of interest.

## Footnotes

^1^Ho, M. Y. (2021). *Is the Character of Forbearance Missing from the List? The Development and Validation of the Forbearance Scale*. (Manuscript in preparation).
